# Clinical Application of Interferon-γ Release Assays for the Prevention of Tuberculosis in Countries with Low Incidence

**DOI:** 10.20411/pai.v1i2.173

**Published:** 2017-01-04

**Authors:** Christoph Lange, Anna M. Mandalakas, Barbara Kalsdorf, Claudia M. Denkinger, Martina Sester

**Affiliations:** 1 Division of Clinical Infectious Diseases, Medical Clinic Research Center Borstel, Borstel, Germany; 2 German Center for Infection Research (DZIF), Clinical Tuberculosis Center, Borstel, Germany; 3 International Health/Infectious Diseases, University of Lübeck, Lübeck, Germany; 4 Department of Medicine, Karolinska Institute, Stockholm, Sweden; 5 Department of Internal Medicine, University of Namibia School of Medicine, Windhoek, Namibia; 6 Global Tuberculosis Program, Department of Pediatrics, Baylor College of Medicine, Houston, Texas, United States; 7 FIND, Geneva, Switzerland; 8 Department of Transplant and Infection Immunology, Saarland University, Homburg, Germany

**Keywords:** Interferon-γ release assays, IGRAs, tuberculin skin test, tuberculosis, *M. tuberculosis*, immunodeficiency, number needed to treat, risk groups, QuantiFERON, T-SPOT.TB.

## Abstract

Despite global efforts to control tuberculosis (TB) the estimated number of people who developed TB worldwide increased to an all-time record of more than 10 million in 2015. The goal of the World Health Organization (WHO) to reduce the global incidence of TB to less than 100 cases per million by 2035, cannot be reached unless TB prevention is markedly improved. There is a need for an improved vaccine that better protects individuals who are exposed to *Mycobacterium tuberculosis* from infection and active disease compared to the current *M. bovis* Bacille Calmette Guérin (BCG) vaccine. In the absence of such a vaccine, prevention relies on infection control measures and preventive chemotherapy for people with latent infection with *M. tuberculosis* (LTBI), who have the highest risk of progression to active TB. During the past decade, interferon-γ release assays (IGRAs) have increasingly replaced the tuberculin skin test as screening tools for the diagnosis of LTBI in countries with a low incidence of TB. Despite recent WHO guidelines on the management of LTBI, the definition of groups at risk for TB remains controversial, and the role of IGRAs for TB prevention in low-incidence countries remains uncertain. We reviewed the scientific literature and provide recommendations for the use of IGRAs for LTBI diagnosis in low-incidence countries. These recommendations are based on the number of patients needing treatment in order to prevent one case of TB. As the positive predictive value of IGRAs for the development of TB is sub-optimal, research must focus on the identification of alternative biomarkers that offer better predictive ability in order to substantially reduce the number needing treatment while improving the prevention of TB and improving the effectiveness of targeted preventive chemotherapy.

**STANDFIRST**

In this article, we discuss the principles and use of IGRAs, and we provide recommendations for their use in countries with low incidence of tuberculosis.

## INTRODUCTION

Tuberculosis (TB) is an airborne infectious disease caused by *Mycobacterium tuberculosis* [1]. The World Health Organization (WHO) estimates that 10.4 million people developed TB and 1.8 million people died from this disease in the year 2015 [2]. Although the annual number of deaths attributed to TB is declining, TB has now surpassed HIV-infection as the leading cause of mortality by a particular microorganism worldwide [3].

Infection with *M. tuberculosis* is transmitted by inhalation of bacilli containing nucleic droplets which are released into the air from patients with pulmonary TB when coughing [4]. Chronic fibro-nodular or fibro-cavitary pneumonia is the predominant clinical manifestation of this disease, and the most common extra-pulmonary manifestation of TB is lymphadenitis. Tuberculosis can potentially involve every organ of the human body [5], and the risk of extrapulmonary manifestations is inversely related to age [6]. The great majority of people infected with *M. tuberculosis* do not develop active disease although it is estimated that one third of the world´s population is infected with *M. tuberculosis*. [2]

In general, the risk for the development of TB increases with exposure to *M. tuberculosis* and along a gradient of immunodeficiency. Inherited susceptibility to mycobacterial infections [7], HIV-infection [8], treatment with tumor necrosis factor (TNF) antagonists [9], and young age [10] carry the greatest risk for the development of TB after exposure.

Despite the enormous burden of TB on many healthcare systems and the dramatic emergence of drug-resistant tuberculosis during the past decade, the WHO recently proposed ambitious targets of reducing the numbers of deaths due to TB from 2015 to 2035 by 95% and the incidence of TB by 90%. Part of the WHO's “END TB Strategy” is the elimination of TB by the year 2050 [11].

In addition to improving treatment outcomes of TB, especially multidrug-resistant and extensively drug-resistant TB, the prevention of TB must be substantially improved to achieve these goals [12, 13]. In the absence of a vaccine that is more effective than *M. bovis* BCG to prevent infection with *M. tuberculosis* and the development of active TB, the prevention of TB relies on 1) early case detection and adequate treatment (treatment as prevention), 2) reduction of transmission by isolation of infectious cases and other infection control measures, and 3) identification and preventive treatment of individuals with latent infection with *M. tuberculosis* (LTBI) [14].

## LATENT INFECTION WITH MYCOBACTERIUM TUBERCULOSIS: A CONCEPT FOR CLINICIANS

Latent infection with *M. tuberculosis* (LTBI) cannot be measured directly. In clinical practice, LTBI is defined by the presence of an *M. tuberculosis*-specific adaptive immune response in the absence of signs and symptoms of active TB [15]. People with LTBI are at risk for developing active TB, and the risk depends on the degree of *M. tuberculosis* exposure and the degree of immunodeficiency. Preventive treatment of persons with LTBI can avert the development of active TB. Treatment may be comprised of one of the following regimens: isoniazid for 6 months, isoniazid for 9 months, rifapentine (weekly) for 3 months (available in most of Europe via an international pharmacy) plus isoniazid, isoniazid for 3 to 4 months plus rifampicin, or rifampicin alone for 3 to 4 months [16]. However, screening for LTBI and preventive treatment must be targeted to high-risk groups to remain cost effective.

The tuberculin-skin-test (TST) has been the standard method for the diagnosis of LTBI for more than a century. The development of this test followed the discovery by von Pirquet in 1907 (the test was later revised by Mendel [17] and Mantoux [18]) that an antigen-specific delayed type hypersensitivity reaction can be provoked by intradermal inoculation of sterile supernatants of *M. tuberculosis* cultures to stimulate *M. tuberculosis*-specific adaptive immune responses in vivo,

In the first study of the TST, von Pirquet made 3 important observations that are still valid [19]: Firstly, neonates with active TB have negative TST results as their adaptive immune system is not yet developed. Secondly, in adolescents (the same is true for adults), the sensitivity of the TST for the diagnosis of active TB is approximately 60%. Thirdly, in countries with a high incidence of TB, one third of the adolescent population has LTBI.

The TST has technical and operational disadvantages which include cross-reactivity to *M. bovis* or non-tuberculous mycobacteria (NTM) after BCG vaccination or NTM infection, requirement of a second visit 48 to 72 hours after application of the skin test, operator variability in test reading, low sensitivity in immunocompromised patients, and a booster effect that can be misinterpreted as a recent conversion. This has led to the development of interferon-γ release assays (IGRAs) as *ex-vivo* alternatives. Two IGRAs are commercially available. Both rely on the principle that blood cells are stimulated *ex vivo* with mycobacterial antigens, which are processed and presented by antigen-presenting cells. T-cells from individuals with prior sensitization recognize mycobacterial antigens, leading to activation and cytokine induction [15]. IFN-γ is commonly used as a readout cytokine. In the QuantiFERON-TB Gold in-tube assay (Qiagen, Hilden, Germany), IFN-γ is detected in supernatants of stimulated whole blood samples using an enzyme-linked immunosorbent assay (ELISA). However, the T-SPOT.TB assay (Oxford Immunotech, Abingdon, UK) quantifies IFN-γ producing cells from isolated peripheral blood mononuclear cells (PBMC) using the enzyme-linked immunospot (ELISPOT) assay principle. Advantages of the IGRAs include shorter stimulation times of 18 to 24 hours only. Moreover, unlike in TST, where stimulation is performed with tuberculin purified protein derivative (PPD), IGRAs use stimuli such as early secreted antigenic target (ESAT)-6 and culture filtrate protein (CFP)-10 that are specific for *M. tuberculosis* due to their absence in *M. bovis* BCG and most NTM. Therefore, IGRAs have a higher specificity to distinguish immunity from *M. bovis* BCG infection or NTM infection from true LTBI or active TB. Moreover, IGRAs are accompanied by negative and positive control stimuli. Indeterminate test results due to failure to react in the positive control (phytohemagglutinin, PHA) have been associated with young age due to immaturity of the immune system [20, 21] and to the extent of immunodeficiency [8]. Similarly, if reactivity in the negative control stimulation is similar to the specific stimulation, this result is considered indeterminate, as the response may not be assigned specificity for mycobacterial antigens, and thereby false positive results are avoided. As compared to the TST, reactivity of the positive control may allow better distinction between true negative results from indeterminate tests, which represents an advantage of IGRAs over the TST [22–25]. Finally, IGRAs have a similar or higher sensitivity compared to the TST, especially when used in immunocompromised patients with T cell-mediated immunodeficiencies such as HIV-infected patients, or stem-cell or solid organ transplant recipients [8, 15, 26, 27]. However, the sensitivity of both tests decreases with increasing severity of immunodeficiency, which also negatively affects the ability to predict progression towards tuberculosis.

Based on the peptide composition, CD4 T cells are the prominent subpopulation that reacts in the 2 commercial assay formats. As an attempt to further increase sensitivity, the “QuantiFERON plus” assay has recently been released which includes an additional tube containing both the CD4 T-cell stimulating peptides derived from ESAT-6 and CFP-10 as well as CFP-10-derived shorter peptides designed to stimulate CD8 T cells. The difference in the level of IFN-γ secretion between the 2 tubes may be considered as a surrogate for CD8 T-cell reactivity. First results with the QFT plus assay may indicate a higher sensitivity for detection of active TB [28], and a better association with risk factors for recent exposure among contacts [28] as compared to the QuantiFERON-TB Gold In-Tube test. Recently, skin-test formats have been further developed that make use of ESAT-6 and CFP-10 as *in vivo* stimuli [29–32]. As expected, this leads to an increase in specificity compared to the conventional TST. The Diaskintest is already widely used in Eastern Europe and Russia, while the c-TB skin test is still under clinical evaluation. Although sensitivity is not likely to improve, the performance of these skin-tests in immunocompromised individuals warrants further study. The properties of the skin-tests and the IGRAs are summarized in Table 1. Common misconceptions and frequently asked questions about IGRAs are stated in Table 2.

**Table 1.** Comparison of immunodiagnostic test characteristics

		Method	Antigen	Time to result	Advantage	Disadvantage
**Skin tests**		Mantoux TST Readout: Skin induration after tuberculin injection (mm)	PPD	48-72h	- inexpensive - recommended for children - largest body of clinical experience	- affected by BCG-vaccination - 2 visits - low sensitivity in immunocompromised - subject to human variability in placement and reading
		Diaskintest^©^: Readout: Skin induration after antigen injection (mm)	ESAT-6 CFP-10	48-72h	- not affected by BCG-vaccination	- 2 visits - sensitivity in immunocompromised not known - available only in Russia and Eastern Europe
		C-Tb skintest^©^: Readout: Skin induration after antigen injection (mm)	C-Tb (rESAT-6 and rCFP-10)	48-72h	-not affected by BCG-vaccination	- 2 visits - sensitivity in immunocompromised not known - not yet commercially available
**IGRAs**	**ELISPOT**	T-Spot.TB^©^: (IFN-γ producing cells/250.000 PBMC) Readout: SFC	ESAT-6 CFP-10 (peptides)	< 24h	- not affected by BCG-vaccination - optimized differentiation between LTBI and TB by comparison of peripheral and local immune response	- no differentiation between LTBI and TB in blood - more elaborate technique compared to QFT-G-IT
	**ELISAs**	QFT-G-IT^©^: (antigen-specific IFN-γ release in whole blood) Readout: IU/ml	ESAT-6 CFP-10 Tb 7.7 (peptides)	< 24h	- not affected by BCG-vaccination - operational test advantages compared to ELISPOT	- no differentiation between LTBI and TB in blood
		QFT-plus^©^: (antigen-specific IFN-γ release in whole blood) Readout: IU/ml	TB1 (ESAT-6, CFP-10) TB2 (TB1 peptides plus short CFP-10 peptides)	< 24h	- not affected by BCG-vaccination - operational test advantages compared to ELISPOT - manufacturer suggests higher sensitivity compared to QFT-G-IT	- no differentiation between LTBI and TB in blood

BCG, Bacille Calmette-Guérin; CFP-10, culture filtrate protein; ESAT-6, early secreted antigenic target; IFN-γ, interferon-γ; PPD, purified protein derivative; PBMC, peripheral blood mononuclear cells; TST, tuberculin skin test.

**Table 2.** Ten common misconceptions about IGRAs

	Misconception	Evidence
**1**	*“IGRAs are tests for the diagnosis of active TB.”*	The pooled sensitivities and specificities for the diagnosis of active TB of the Quantiferon-Gold-in tube test and the T-Spot-TB test were 80% (95% CI 75– 84%)/ 79% (95% CI 75–82%) and 81% (95% CI 78–84%)/59% (95% CI 56–62%) In a recent systematic-review and meta-analysis [33]. The diagnostic accuracy is higher, when IGRAs are performed on extrasanguinous fluids, e.g. CSF, ascites, BAL [33, 34]. Standard IGRAs performed with cells from the peripheral blood should not be used for the diagnosis of active TB.
**2**	*“A negative IGRA test result excludes active TB.”*	In active TB approximately 20 % of patients have a negative IGRA test result [35].
**3**	*“IGRAs can safely exclude the progression to active TB because of a very high negative predictive value.”*	While the negative predictive value of IGRAs is very high when healthy contacts are evaluated, the negative predictive value is low in HIV-infected hosts. More than 50 % of HIV-infected individuals who later developed active TB in low incidence countries of TB had a negative test results in both IGRAs at the time of screening [8].
**4**	*“IGRAs are better than the TST to predict progression to active TB in immunocompromised patients.”*	In the largest prospective multicenter observational cohort study that evaluated the role of IGRAs and the TST as predictors for progression to active TB in immunocompromised hosts, prediction of disease progression by IGRA was not superior to the TST [8].
**5**	*“The higher the concentration of IFN-*γ *in the QFT-G-IT test or the number of spot-forming cells in the T-SPOT.TB test, the greater the risk for the progression to active TB.”*	The cut-offs for test-positivity provided by the manufacturers of the QFT-GIT and the T-SPOT.TB test have been validated in at least one prospective study including more than 5000 close contacts of patients with active TB. In contrast to previous smaller studies, in this multinational study in Europe, there was no correlation between the magnitude of a positive test and the risk for progression to tuberculosis [36].
**6**	*“After treatment initiation for active TB presence and magnitude of IGRA responses reflect the response to anti-TB therapy, thus IGRAs can be used for treatment monitoring.”*	In those studies that evaluated possible relationships between IGRA responses and treatment-monitoring parameters no clear correlation could be found [37–40].
**7**	*“Positive IGRA responses that develop after anti-TB vaccination are an indicator for protective immunity against TB.”*	In a large placebo controlled prospective randomized trial evaluating a novel MVA-Ag85 vaccine, *M. tuberculosis* specific immune responses could be detected by IGRA in the group of MVA-Ag85 vaccinated children, but not in the placebo group. During follow-up, the TB incidence was similar on the MVA-Ag85 vaccine and in the placebo arm, suggesting that IGRAs are not correlate markers of protective immunity against TB [41].
**8**	*“Health-care workers in low-incidence countries of TB should undergo regular IGRA screening.”*	In 3 recent studies where health-care workers were tested by IGRAs in low-incidence countries of TB and followed for > 2 years not a single case of TB occurred among > 1500 IGRA positive healthcare workers in the absence of preventive chemotherapy [42–44]. The risk of healthcare workers for the development of TB is low, unless a specific intensive contact to a TB patients occurred. Regular screening is not justified.
**9**	*“A positive IGRA result carries the same risk for the development of tuberculosis in different groups of patients or healthy individuals.”*	The positive predictive values of IGRAs and the number needed to treat to prevent one case of tuberculosis based on IGRA results show substantial differences among different groups of individuals. In persons with HIV infection and ongoing viral replication [45] or in small children who are household contacts [36], the number needed to treat to prevent one case of TB is approximately 10, thus IGRA screening followed by preventive chemotherapy is highly effective. In persons from low-incidence countries of TB with chronic renal failure 1/ 4 persons has a positive IGRA test result but the risk for TB is very low [8], Regular screening and preventive chemotherapy is not indicated in this group.
**10**	*“Indeterminate test results are always caused by immunodeficiency.”*	While failure of the positive control is the most common cause of an indeterminate IGRA test result, failure of the negative control may also be a cause of an indeterminate test result [8], most commonly in persons with autoimmune disorders (e.g. Systemic lupus erythematodes).

### How well is the progression to tuberculosis predicted by IGRAs in countries with a low incidence of TB?

Recommendations to use IGRAs in countries with a low incidence of TB are restricted to asymptomatic individuals who have an increased risk for progression towards tuberculosis, and for the purpose of guiding preventive chemotherapy. According to WHO guidelines, individuals at risk include close contacts of patients with active TB, migrants, health care workers, patients with diabetes, and immunocompromised patients with HIV infection, chronic renal failure or receiving immunosuppressive drug therapy, treatment with TNF antagonists or other biological therapeutics [46]. Although IGRAs have a high specificity and good sensitivity for diagnosing latent or active infection with *M. tuberculosis*, several studies from countries with low incidence of TB have shown that only a very small percentage of individuals with positive test results develop active TB, and the risk for progression differs among the groups considered at risk [8, 33, 36, 42, 43, 47–50]. Moreover, patients with severe immunodeficiency who develop TB may have had negative test results at the time of screening, which emphasizes that the negative predictive value is not sufficient in this situation [8]. The ability of IGRAs to predict progression towards TB in various risk groups from low-incidence countries was extracted from recently published key studies with sample sizes of more than 1 000 individuals, and performance characteristics are summarized in Table 3. We have also included a study in migrant contacts to show comparative data for this risk group. Although the selection of studies was not based on a formal systematic review, the focus on analyses with large sample size mostly derived from multicenter analyses allows for a representative estimation of IGRA performance in current clinical practice. Although the risk for the development of TB is highest in the first year following contact with *M. tuberculosis* and the follow-up time in these studies was between 2-5 years, some individuals may develop TB at a later time. Therefore, results from these studies may underestimate the long-term risk for the development of tuberculosis.

**Table 3.** Numbers needed to treat to prevent one case of TB in people with a positive IGRA or TST test result in different risk-groups in low incidence countries

Study	Country	Population	LTBI test	LTBI positive (%)	Number followed longitudinally	TB cases incident	Sensitivity %	Specificity %	PPV %	NPV %	NNT
**Zellweger et al [36]**	Europe	contacts	QFT-G-IT	1067 (27.4%)	3425	20	85.0	74.0	1.9	99.9	**37**
			T-SPOT.TB	299 (26.6%)	1061	4	50	73.6	0.7	99.7	**37**
**Geis et al [47]**	Germany	contacts	QFT-G-IT^c^	306 (19.3%)	254	6	100	n.d.	2.5	n.d.	**34**
**Sloot et al [48]**	Netherlands	contacts	TST/QFT-G-IT	739 (15.5%)	4716	17	76.5	85.8	1.9	99.9	**89**
		Close contacts	TST/QFT-G-IT		1622	10	90	79	2.6	99.9	**30**
**Kik et al [51]**	Netherlands	Migrant contacts	TST^d^	339	339	8	87.5	n.d.	3.8	n.d.	**26**
			QFT-G-IT	178	327	8	62.5	n.d.	2.8	n.d.	**36**
			T-SPOT.TB	181	299	8	75	n.d.	3.3	n.d.	**30**
**Hermansen et al [49]**	Denmark	Mixed^a^	QFT-G-IT	1703 (10.7%)	15980	40	50	88.7	1.32	99.9	68^e^
**Sester et al [8]**	Europe	Immuno-compromised^b^	TST	212 (14.1%)	1404	6	50	86.2	1.5	99.8	**50**
			QFT-G-IT	239 (15.6%)	1342	4	50	84.1	0.9	99.8	**80**
			T-SPOT.TB	266 (17.7%)	1310	6	50	81.3	1.3	99.7	**64**
		HIV only	TST	55 (8.7%)	626	6	50	93.7	7.1	99.5	**14**
			QFT-G-IT	83 (13.1%)	621	4	50	92.1	3.9	99.6	**26**
			T-SPOT.TB	101 (15.9%)	561	6	50	89	4.7	99.4	**21**
		HIV positive HIV load	TST	24 (8.1%)	291	6	50	92.6	12.5	98.9	**8**
			QFT-G-IT	25 (8.4%)	289	4	50	91.9	8	99.2	**13**
			T-SPOT.TB	31 (10.4%)	255	6	50	88.8	9.7	98.7	**10**
**Schablon et al [50]**	Germany	HCW	QFT-G-IT	317 (8.3%)	3823	0	0	91.7	0	100	**n.a.^f^**
**Slater et al [43]**	USA	HCW	QFT-G-IT	853 (9.3%)	9153	0	0	90.7	0	100	**n.a.^f^**
**Dorman et al [42]**	USA	HCW	TST	125 (5.2%)	2418	0	0	94.8	0	100	**n.a.^f^**
			QFT-G-IT	118 (4.9%)	2418	0	0	95.1	0	100	**n.a.^f^**
			T-SPOT.TB	144 (6.0%)	2418	0	0	94	0	100	**n.a.^f^**

^a^individuals included contacts, patients prior to TNF antagonist treatment, and TB suspects;

^b^individuals included patients with chronic renal failure, patients prior to TNF antagonist treatment, solid organ- and stem cell transplant recipients, and HIV infected patients; PPV, positive predictive value; NPV, negative predictive value; NNT, number needed to treat to prevent a case of TB; LTBI, latent infection with M. tuberculosis; TST, tuberculin skin test; QFT-G-IT, QuantiFERON gold in tube; HCW, health-care workers; PPV and NPV were calculated from all individuals with and without preventive chemotherapy;

^c^only IGRA-positive individuals were included and followed longitudinally;

^d^only TST positive individuals were included and followed longitudinally;

^e^assumption no case of TB in IPT treated individuals, 35% INH coverage among test positives;

^f^no cases of active TB.

The number needed to treat (NNT) to prevent one case of TB is a clinically useful parameter based on test results to guide preventive chemotherapy in a given risk group. It is based on the absolute risk reduction (1/absolute risk reduction), and is calculated from the difference in the number of TB cases found on follow-up in individuals with positive test results between groups with and without preventive chemotherapy. Recently, several large studies with more than a 1000 individuals each have been published that allow a robust estimation of the NNT for various groups considered at risk. A large European multicenter study performed in the TuBerculosis Network European Trialsgroup (TBNET; www.tb-net.org) included 4513 close contacts of patients with active tuberculosis who were screened with either QFT-G-IT or the T-SPOT. TB assay [36]. The diagnosis of tuberculosis was overall a rare event with only 24 incident cases. The incidence of tuberculosis was substantially lower in contacts with preventive chemotherapy, indicating that treatment was highly effective. Also, the negative predictive value was nearly 100%, emphasizing that tuberculosis is rare in IGRA-negative contacts. Nevertheless, the positive predictive value of a positive IGRA was low (1.9 and 0.7 for QFT-G-IT or the T-SPOT.TB), which shows that the majority of contacts with positive IGRA did not develop TB. Taking these results together and based on this study, the NNT to prevent one case of TB is 37 for QFT-G-IT and 37 for the T-SPOT.TB (Table 3) [36]. Interestingly, these NNTs are very similar to those derived from another contact-tracing study involving 1579 subjects in Germany [47], where the NNT for the QFT-G-IT was 34. Finally, in a large Dutch study, where 4716 contacts were screened using a TST and/or IGRA, the NNT reached 89. Interestingly, if screening was restricted to close contacts, this led to a substantial decrease in the NNT to 30 (Table 3) [48].

Together these findings illustrate that the NNT among contacts is in the range of 30 to 37, and that this NNT-range may be achieved if screening is focused on close contacts (Table 3). The NNT may be considerably higher if individuals from several risk groups are estimated together. This is illustrated by a nation-wide study from Denmark which included 15 980 individuals after contact tracing, screening prior to TNF antagonist treatment, or suspected of having TB. In this study, 40 incident TB cases were observed, and the PPV of a positive QFT-G-IT was 1.32 only with an estimated NNT of 68 [49]. Interestingly, this corresponds to the higher NNT calculated from a large multicenter study among immunocompromised patients including HIV-infected individuals, patients with chronic renal failure or rheumatoid arthritis, or stem-cell and solid organ transplant recipients [8]. In the combined analysis of all patients, the NNT was 50 for the TST, 80 for the QFT-G-IT, and 64 for the T-SPOT.TB assay. It should be noted that for immunocompromised patients the NNT was higher with the use of IGRAs than with the use of TST. In addition, TB cases also occurred in patients who tested negative with IGRA/TST and were almost exclusively found among HIV-infected patients; all HIV-patients developing TB had ongoing HIV replication [8, 45]. Thus, targeted preventive therapy for immunocompromised hosts in low TB incidence countries is most effective in HIV-infected patients with detectable viral replication (NNT of 8 for the TST, 13 for the QFT-G-IT, and 10 for the T-SPOT.TB assay, (Table 3) [45, 52].

Health-care workers represent another group of individuals where serial monitoring has been recommended due to a presumed higher risk for disease progression. However, as emphasized by 3 large studies from Germany and from the United States involving single or serial screening of more than 15 000 individuals, no case of tuberculosis occurred (Table 3) [42, 43, 50], which may suggest that serial screening of health-care workers should not be performed and should be restricted to personnel after close contact with a patient with active TB. Alternative definitions of IGRA conversion and different cutoff points for different risk populations should be developed [53]. These would need to be evaluated in large data sets and modeling studies [43, 54].

Finally, screening is recommended for migrants. A study from the Netherlands analyzed progression towards TB in approximately 300 migrant contacts of cases with active TB [51]. Although progression towards TB in those with negative tests was not reported, the estimated NNT of migrants who had active TB contacts was similar to other contacts with active TB cases (26 for the TST, 37 for the QFT-G-IT, and 30 for the T-SPOT.TB test) (Table 3).

Taken together, a comparison of the NNTs of these studies from countries with a low incidence of TB indicates that the risk for progression differs from one group to another. Moreover, as illustrated by the lowest NNT in close contacts or migrant contacts, or in HIV-infected individuals with ongoing HIV-replication, targeted screening of individuals with additional, clinical risk factors can lead to a substantial decrease in screening load and unnecessary preventive chemotherapy. Similarly, in other groups previously considered at risk such as health-care workers or patients with chronic renal failure, where general LTBI testing was recommended, screening [46] and treatment may now be focused on individuals with additional risk factors.

The benefits of preventive chemotherapy also have to be weighed against age-related drug-associated hepatoxicity [55, 56], and the complexity of such trade-offs might be best judged by data-driven algorithms [57]. Table 4 provides recommendations based on available evidence for the NNT regarding the use of IGRAs and preventive chemotherapy in countries with low incidence of TB.

**Table 4.** Recommendations for the use of IGRA-testing* and preventive chemotherapy against active tuberculosis in low-incidence countries according to the number needed to treat to prevent one case of TB.

Recommended	Conditionally recommended	Not recommended
People living with HIV (especially with ongoing viral replication)^a^	Solid organ transplant recipients^c^	Health-care workers^d^
Child and adult contacts of pulmonary TB cases^a^	Stem- cell transplant recipients^c^	Illicit drug users^e^
Migrants originating from high incidence countries^a^	Patients with chronic renal failure^c^	Homeless people^e^
Patients receiving tumor necrosis factor (TNF)- antagonist therapy^b^	HIV-negative patients with other immunodeficiencies not mentioned above^c^	Patients with diabetes mellitus^e^
	Patients with silicosis^c^	Prisoners^e^
		People with alcohol abuse^e^
		Tobacco smokers^e^
		Underweight people^e^

^a^number needed to treat to prevent a case of TB of < 50;

^b^number needed to treat to prevent a case of TB of > 50 but justified by severity of TB disease;

^c^number needed to treat to prevent a case of TB of > 100; at least one additional risk factors for LTBI should be present;

^d^number needed to treat to prevent a case of TB of >> 100 or

^e^not known but perceived to be high; unless other factors leading to strong or conditional recommendations are present;

*the best method is to combine IGRA/TST results with a risk/benefit assessment performed by algorithms [57].

Screening for LTBI is currently also recommended and frequently used in other patients considered at risk such as patients prior to TNF antagonist treatment or receiving other immunotherapies for neoplastic, hematologic, rheumatologic, dermatologic, and other conditions. Similar studies with large sample sizes are also needed to define the NNTs for these groups. Additional information for the use of IGRAs and preventive chemotherapy in countries with high or intermediate incidence of TB, where IGRAs are generally not recommended to screen for LTBI, is provided in an appendix.

### Are the proposed IGRA cutoffs valid?

Based on the comparison between healthy controls and patients with culture-confirmed TB, the following cutoffs were established for the 2 commercially available IGRAs: a positive T-SPOT.TB result is defined as antigen-specific induced IFN-γ secretion of more than 5 spot forming cells (SFC) per 250 000 PBMC after subtraction of the negative control reactivity [58], with a borderline zone between 5 and 7 SFC defined for use in the United States. The result of the QFT-G-IT assay is defined as positive above a cutoff of 0.35 IU IFN-γ/ml whole blood after subtraction of the nil control [59, 60]. For the purpose of sensitivity, low cutoffs were assigned in order not to miss any cases.

Reproducibility studies have identified some inter-test variability [57]. This may be due to variability in the individual immune function. Moreover, variations might be due to the time of blood collection [61], delay or differences in processing, or duration of incubation. Important immunological sources might be inadequate disinfection before phlebotomy or previous TST boosting.

These variations have most impact if quantitative IGRA results are close to the cutoffs. Serial examinations showed conversion rates between 2.8% and 8.3% and very high reversion rates between 37.3% and 64.8% [42, 43, 50, 62]. This has led some authors to propose borderline definition for positivity to avoid unnecessary chest-X-ray or preventive treatment [44].

Although both IGRAs give quantitative results, clinical decisions are based on qualitative results. One study suggested that an increase in the cutoff value may increase the positive predictive value to predict disease progression and decrease the NNT to prevent a single case [47]. However, these results were not confirmed in a larger study [36], where the currently recommended cutoffs for test positivity provided the best discrimination between those who did and did not progress to active tuberculosis [36]. While these cutoffs appear to be optimal for the TB risk evaluation among contacts, it must be acknowledged that a larger proportion of patients with immunodeficiencies who progress to TB, may have negative IGRA test results at the time of screening [8].

### Are IGRAs tools to monitor tuberculosis treatment responses or surrogates for vaccine effectiveness?

The IGRAs measure the response of circulating effector T-lymphocytes to viable and dormant mycobacteria [63], and results are assumed to correlate with the antigenic load present in the body [37, 39, 64]. Treatment and decrease of antigenic load therefore should result in a decrease in IGRAs reactivity, which could conceivably be used for treatment monitoring [64–66].

### Active TB

Few studies have tried to correlate the changes in IFN-γ responses in IGRAs with established markers of treatment response or outcome [37, 39, 40, 67]. Overall, no clear correlation of IGRA response with treatment-monitoring parameters could be found. This is probably attributable in part to significant within-subject variability in sequential measurements due to both exogenous (eg, relating to test procedure) and endogenous (eg, co-infections, time from exposure) factors [68].

### LTBI

While there are established markers for treatment monitoring in active TB (ie sputum and smear conversion), no biomarker is currently available for the monitoring of latent TB treatment success. By definition, no mycobacterial product is identifiable in LTBI with current techniques, and therefore a reliance on immune-based tests for monitoring is even greater [69]. However, a significant change using an IGRA has been proven difficult to detect probably because (i) IFN-γ is produced in low quantity and mostly in close proximity to the antigen [70–71]; (ii) changes in IFN-γ response independent of treatment have been observed, which might reflect self-clearance or transition of bacteria into dormancy [72]; (iii) an initial increase in IFN-γ response due to increased antigen stimulation from destruction of mycobacteria can be observed early in treatment [73]. Overall it can be stated that IGRAs do not appear to be a promising biomarker for treatment monitoring in LTBI.

Recently IGRAs were used to describe vaccine-induced cytokine immune responses following a MVA-85 vaccine-specific stimulation in neonates [41]. In this study, induction of a vaccine-specific IGRA response did not correlate with improved capacity to control mycobacterial growth and to protect individuals from active TB.

### Special considerations in children

As in adults, IGRAs cannot differentiate between LTBI and active disease in children. Preventive therapy programs optimize cost-effectiveness by targeting populations at greatest risk of TB infection and disease. Prior to initiating preventive therapy, children must complete screening to exclude TB, but confirmation of LTBI is not mandatory. Among young household contacts in TB high-burden settings (in whom the risk of LTBI may be > 40% [74]), it is most cost-effective to provide preventive therapy to all child contacts younger than 5 years without testing for LTBI [75]. This approach may also be reasonable for child close contacts in countries with low incidence of TB. However, in most TB low-burden settings, documentation of a positive IGRA or TST result guides the use of preventive therapy in child contacts [76–78].

Microbiological confirmation of child TB is rare due to the challenges of sputum collection, the paucibacillary nature of the disease, and the low yield of diagnostic tests [79]. A systematic review estimates that 75% of children receive TB treatment based on bacteriologically unconfirmed, clinically diagnosed TB [80]. IGRAs are commonly used in children living in upper income, low TB-burden countries, and the results influence clinical decision making [81]. Pragmatic use of IGRAs and TST in children have emerged through clinical practice and informed US guidelines [77]. In low-risk, BCG-vaccinated or NTM-exposed children (in whom positive TST results are expected due to adaptive immunity against other mycobacteria), high specificity is desired which makes the use of IGRAs preferable. In young or immunocompromised children with significant risk of TB progression, testing strategies optimize sensitivity by using both an IGRA and a TST; a positive result with either test is considered evidence of LTBI. Guidelines in the United Kingdom recommend TST as the first test of LTBI, with use of IGRAs only in children with a negative TST or at times when the TST is unavailable or impractical [78]. Because several studies have shown that IGRAs are more likely to be indeterminate or negative in children younger than 5 years, current US CDC guidelines recommend the use of TST rather than IGRA in children younger than 5 years [76]. The WHO preferentially recommends the TST [82] and recommends against the use of IGRAs in TB high-burden settings [83].

## CONCLUSIONS

As the risk for tuberculosis depends on host susceptibility and pathogen exposure, the definition of risk groups for targeted LTBI screening varies substantially over time and is dependent on the geographic region.

While health-care workers in the United States and Western Europe used to be considered a high-risk group for TB as the prevalence of the disease was thought to be high, recent studies have shown that the risk of TB for these workers is low, justifying screening by IGRAs only when a special exposure has occurred, similar to household contacts [42–44]. In people with HIV-infection in countries with a low prevalence of TB, the risk for TB is dramatically reduced in patients who experience sustained undetectable levels of viral replication [45, 84]. It is presently unclear whether all HIV-infected patients in these countries should be regularly screened by IGRAs or whether screening should be restricted to the minority who are antiretroviral therapy (ART) naive, are failing HIV-therapy, or have had recent known *M. tuberculosis* exposure. More than 25% of patients with chronic renal failure from countries with low incidence of TB have positive IGRA responses despite no substantially increased risk for the development of TB [8]. These patients should be screened for LTBI in high-incidence countries where they have an increased risk for *M. tuberculosis* exposure. They should not be routinely screened in low-incidence countries where a positive test result in these patients is not related to a substantial risk for the development of tuberculosis.

In order to provide evidence-based recommendations that are valid for specific geographic regions, cohorts of putative risk groups for TB must be followed longitudinally to evaluate the performance of immunodiagnostic testing, the risk for tuberculosis, and the effect of preventive chemotherapy in different groups and regions. Consensus must be reached on the NNT to prevent one case of tuberculosis. This consensus should guide national and international management recommendations. The recommendations that are provided in this review (Table 4) allow guidance using sub-group stratified best evidence.

Without knowledge about the NNT to prevent a case of tuberculosis in different groups and regions there is both the risk of undertreatment and overtreatment and lack of acceptance of preventive therapies [85].

In order to achieve the goal of TB elimination in low-burden countries, prevention of TB must be improved substantially. Without a vaccine that protects individuals exposed to *M. tuberculosis* better than *M. bovis* BCG, TB prevention strategies must rely on infection control measures and preventive chemotherapy for those individuals with LTBI, who have the highest risk for the progression to active TB [86]. Unfortunately, none of the presently available tests for LTBI offer a high prognostic value. In countries with low incidence for TB, immunodiagnosis is presently the most effective measure to identify people with LTBI who should receive preventive chemotherapy to avoid progression to TB. Considering multiple factors when determining individual risk of future TB is attempted in adult algorithms such as the Online TST/IGRA interpreter, which can likely maximize the prognostic capability of available and future diagnostic tools [57].

A recent study in a large cohort of adolescents from Africa has identified a 16 gene transcriptomic signature from the peripheral blood that was highly predictive for the development of TB and that appears superior to IGRAs in predicting disease development [87]. Promising results were validated in 2 independent African cohorts suggesting that the NNT to prevent one case of TB could be lowered to < 10 by transcript analysis. However, it is presently unclear how well this method will predict the development of TB in individuals from different risk groups in countries with low incidence of TB and how specific such a test will be. Large multinational and multicohort studies are needed to address this question.

While using IGRAs as screening tools for providing preventive chemotherapy for people with positive test results is effective for the individual risk reduction for TB in people from high-risk groups, the effect of screening programs on the overall incidence of TB in low-incidence countries is likely to be low. At times of high global mobility, elimination of TB in low-incidence countries will not be possible by an IGRA-based prevention approach as long as a steep gradient of the burden of TB continues to exist between different geographic regions of the world [88].
